# A dedicated website for cancer subjects, the nutritional support study: preliminary results

**DOI:** 10.3332/ecancer.2011.228

**Published:** 2011-12-20

**Authors:** P Gnagnarella, AM Misotti, L Santoro, D Akoumianakis, G Milolidakis, F De Lorenzo, C Lombardo, R Sullivan, G Mcvie

**Affiliations:** 1 Divisione di Epidemiologia e Biostatistica, Istituto Europeo di Oncologia, Milan, Italy; 2 Center for Technological Research of Crete, Technological Education Institution of Crete, Greece; 3 Federazione Italiana delle Associazioni di Volontariato in Oncologia, Rome, Italy; 4 IRCCS Azienda Ospedaliera Universitaria San Martino – IST – Istituto Nazionale per la Ricerca sul Cancro, Genoa, Italy, Organisation of the European Cancer Institutes, Brussels; 5 Kings College London, and Project Manager of EuroCancerComs- FP7; 6 Direzione Scientifica, Istituto Europeo di Oncologia, Milan, Italy

## Abstract

**Background:**

The Internet has become a widely used resource for information on cancer and for support. As part of the EuroCancerComs project (www.eurocancercoms.eu), an intervention study has been designed. The study aims to help patients with cancer providing an Internet “space” where to find information about nutritional care.

**Methods:**

The study consists of a randomized 6-month intervention. The website (www.supportonutrizionale.it) hosts a contents area, prepared according to guidelines and recommendations, a forum and a blog. Subjects have been randomly allocated in intervention (IG) and control group (CG). IG has a free access to the website and it is involved in live activities, discussions and examinations. CG receives the same information by e-mail, without having access to the website. Three questionnaires are used to evaluate the effectiveness of the approach, concerning quality of life (QoL), psychological status and nutrition facts.

**Results:**

Since the study startup, 191 subjects have been screened, and 58 (30%) have been randomized. Participants in both groups are mainly females, married and have at least a high school education level. Participants experienced a high psychological distress for 27% of IG and 33% of CG considering the four classes of scores at the baseline. Regarding QoL, a low “role functioning” score for IG and “emotional functioning” and “social functioning” scores for both groups are reported, while “fatigue” and “nausea and vomiting” respectively for IG and CG are the worsened symptoms compared with reference values. Considering the nutrition facts questionnaire, subjects showed a medium-high score profile and the worst scale regards “Nutrition and cancer knowledge”. From the beginning of the study, a total of 48 actions have been registered, including votes to contents, comments and forum messages.

**Conclusion:**

The Internet has made possible the new forms of interaction and knowledge, and it is likely to become essential to gain access to health information. The results of this randomized intervention may help in the evaluation of the efficacy of these interventions in cancer setting.

## Background

During the past decade, the Internet has changed the mass communication, and jointly with the associated technologies is the most popular source of information on health [1–4]. It can contribute to disease prevention and health promotion in a number of contexts, crossing boundaries (or fields of expertise) and making new grounds for turning experience [5,6]. The Internet has become a widely used resource for information on cancer [7,8] and for support [7]. In the same time, the use of Internet addresses the social inequality in the Internet access issues [7,9]. Randomised studies suggest that introducing Internet-based support to cancer survivors may result in significant positive outcomes regarding social support, competence in finding information [10], depression [11] and self-perceived health status [12], even if the quality of life of patients with cancer has not been shown to be improved [10–12].

In parallel, also Internet–delivered interventions may be effective in health education. It is not surprising that these interventions were found to be effective in changing health-related behaviours, such as nutrition education, physical activities and weight reductions [13,14].The western dietary and lifestyle pattern are responsible for major chronic diseases [15], and their correction could influence the rate of cancer progression, improve quality of life and overall survival [16–19].

As part of the EuroCancerComs project (www.eurocancercoms.eu [20]), a coordinating action of the European Commission aiming to establish one efficient communication for patients with cancer and caretaker from clinical researchers, scientists and physicians, in collaboration with the *Organisation of European Cancer Institutes* (OECI; www.oeci-eeig.org), the Alleanzacontroilcancro (ACC; www.alleanzacontroilcancro.it), the *Italian Association for cancer patients, their families and friends* (AIMaC – Associazione Italiana dei Malati di Cancro; www.aimac.it) and the *Italian Federation of Volunteer-Based Cancer Organizations* (FAVO – Federazione italiana delle Associazioni di Volontariato di Oncologia; www.favo.it) we designed an intervention study to help patients with cancer. We hypothesised that providing a protected Internet “space” for patients with cancer where to find information about nutritional care and a space where they can interact with experts and/or others participants, would positively affect their knowledge and their status as measured by three instruments: the quality of life, the psychological distress inventory and a questionnaire on nutrition facts. The present paper reports the study design and the preliminary results.

## Methods

### Participants – recruitment – randomization

This intervention study accrued patients with cancer searching for nutritional advice on Internet web sites of the study partners, starting in March 2011. During the first months the following websites published periodically information about the intervention study: mainly the AIMaC website, with a section dedicated to the nutritional problem of cancer patients [21,22] and the FAVO websites; the Facebook and Twitter pages of AIMaC, the European Institute of Oncology of Milan (IEO, Istituto Europeo di Oncologia) and the Umberto Veronesi Foundation (Fondazione Umberto Veronesi). In addition, printed leaflets have been distributed at the IEO.

**Figure 1:**
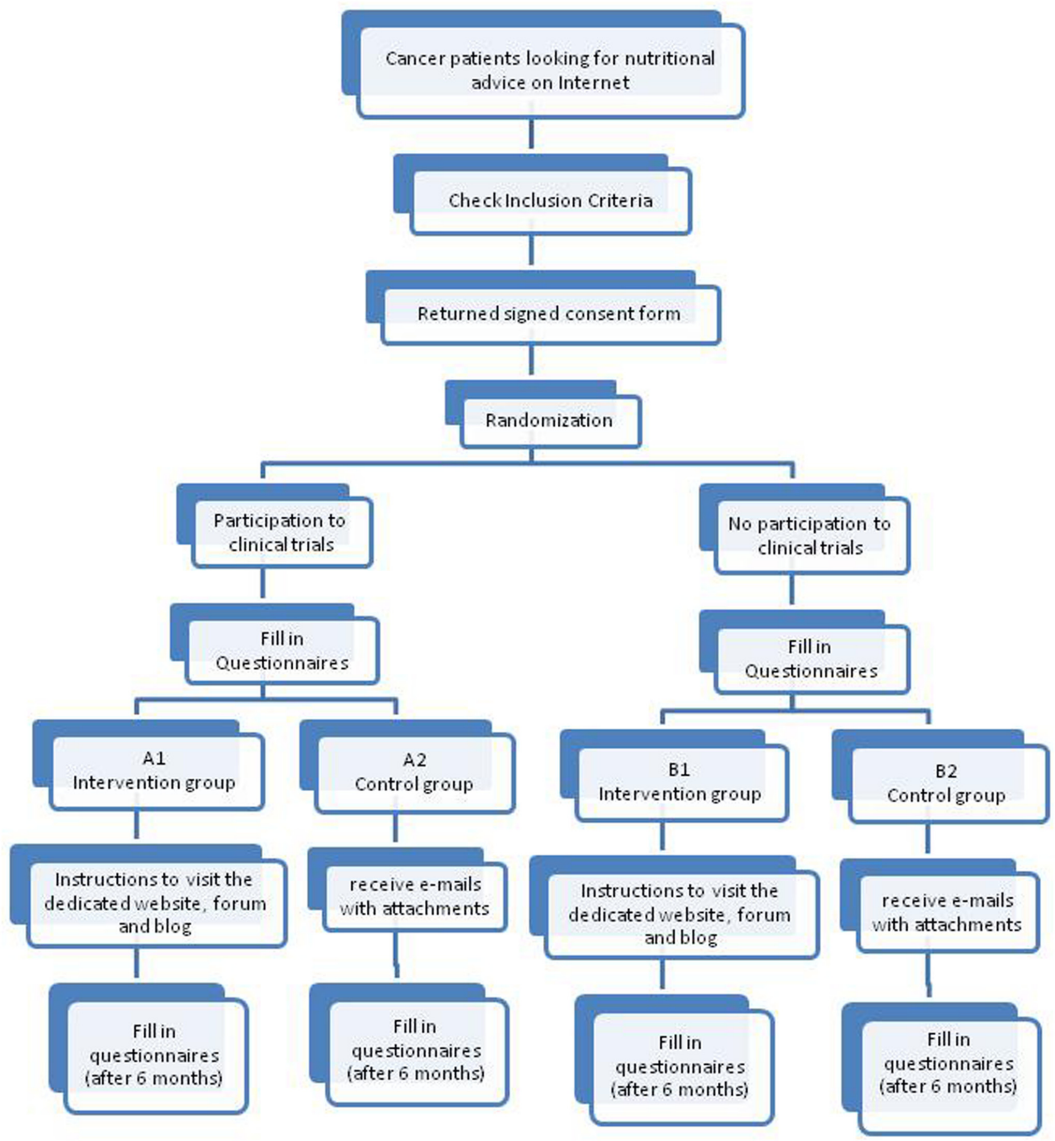
Study design.

Subjects have been invited to take part in a 6-month randomized intervention study [23,24]. Screening before randomization has been conducted to ensure that the study population meet the inclusion criteria as reported in Table 1. Two-arm randomization list has been created by using a computer–generated scheme located at TENALEA website (http://tenalea.net), stratifying subjects according to their participation to previous clinical trials. Subjects have been allocated to one of the two study groups: intervention (IG, A1 and B1) and control group (CG; A2 and B2) (Figure 1).

The study protocol has been approved by the Independent Ethical Committee of Eurocancercoms.

### Intervention

**Figure 2:**
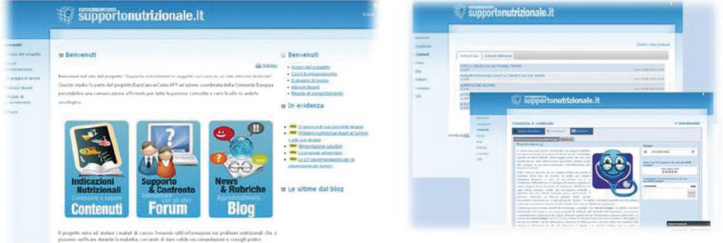
Supporto Nutrizionale Homepage and Baseline Contents (www.supportonutrizionale.it).

The intervention group (IG) was intended as a dedicated web site (Figure 2) where to find information, to interact with experts and other participants and to ask for specific questions. All website sections and functions have been set up with the collaboration of the Technological Educational Institution of Crete. The website hosts a contents area, a forum and a blog beyond some pages dedicated to information about the study and the working groups. All baseline contents, part of the weekly contents and in-depth blog examinations have been prepared using national and international documents and recommendations [21,25–29]. The contents have been delivered weekly on the web space and they refer to three main topics: how to manage the nutrition and eating problems during cancer symptoms; how to control the weight loss and maintain the body weight; guidelines for healthy eating habits. Participants have access to a dedicated discussion forum where they can cultivate social bonds, share opinion, discuss on the topics above or on other topics that may arise from discussions. They could also interact and ask questions to a group of experts established for the study. It consists of an oncologist, a patient representative, a pharmacist, a psychologist, a dietician and an oncologist expert in palliative care. In addition, some interactive activities have been planned for the IG (polls, chat room to talk to the expert).

**Figure 3:**
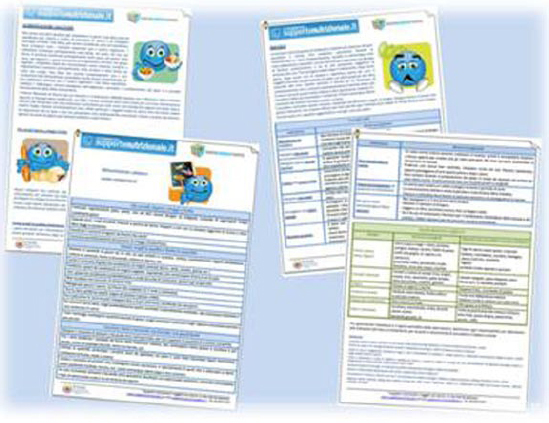
Baseline and weekly contents in Adobe format (Acrobat Reader)

The control group (CG) do not have access to the web space dedicated to the study, but they receive by e-mail (in Adobe format, pdf) (Figure 3), weekly over the study period, documents containing the same information available on the Internet web space for the intervention group. They are not involved in the web space activities (forum, discussion, polls, etc.), although they are free to navigate on Internet without any indication and instructions. A dedicated telephone line and e-mail have been identified to answer questions, to clarify study aspects and to help and to support participants.

### Questionnaires

Three instruments, established to evaluate the effectiveness of the approach, have been administered at the baseline (pre-test) and will be re-administered at the end of the intervention study after 6-month (post-test) to all subjects (IG and CG), to assess the changes over time from the participants.

Psychological Distress Inventory (PDI): a validated self-administered questionnaire to measure anxiety and depression, developed by Morasso [30].

Quality of Life questionnaire (QoL): an instrument developed by the European Organization for Research and Treatment of Cancer (EORTC) to assess Quality of Life in clinical trials and clinical practice [31].

Questionnaire on nutrition facts: this instrument developed by the study team, contains 20 items inquiring about: general facts on healthy eating habits; specific information on how to manage the nutrition and eating problems during cancer symptoms and food consumptions for vegetable and fruits, meat and alcoholic beverages [see Appendix 1]. This instrument has been evaluated by an expert panel and has been tested for interest, readability and comprehension on a group of patients with cancer frequenting IEO (pilot study).

In addition, all participants have been required to fill in at the baseline a questionnaire regarding their socio-demographics characteristics and information about the tumour (site, year of diagnosis, treatment characteristics).

### Statistical analysis

The study in its original form was planned to determine whether providing online information to patients and giving them opportunities to interact, could result in a significant change about their nutritional knowledge, quality of life and psychological status (measured by the three questionnaires). The study requires a final number of 252 subjects to detect a 6-month difference on patients’ knowledge of four points between IG and CG (10% improvement than the expected value at baseline), with a type I error rate of α=5%, 80% power and a 20% dropout rate. The sample size calculation was planned to test the primary endpoint with non–parametric (Mann–Whitney-Wilcoxon) test. The present work, however, shows preliminary results on the first 20% of randomized patients and no analysis has been performed on the original primary endpoint. Moreover at this recruiting level, the two randomized groups might be not balanced yet. For this reason the group characteristics at baseline have been compared by a chi-square test for categorical variables and by a t-test for the continuous ones. In general both descriptive and analytic statistics have been applied to present the preliminary results.

The subject’s participation is measured for each participant by the number of accesses per day, week and month, number of comments/questions posted on the web site, and the active participation to any live activities (i.e.: discussion, pools).

The PDI questionnaire consists of 13 multiple-choice questions (‘not at all’, ‘a little’, ‘quite a bit’, ‘much’, ‘very much’), rated from one to five. Following the PDI manual [32], the global score has been evaluated overall and also divided in four classes. The last class, with a score more than 35, includes those subjects with a high psychological distress.

The QoL (QLQ-C30) incorporates nine multi-item scales: five functional scales (physical, role, cognitive, emotional, and social); three symptom scales (fatigue, pain, and nausea and vomiting); and a global health and quality of life scale. Several single-item symptom measures are also included [33]. The scores have been compared against published data, by using the data for comparable groups of patients published in the EORTC QLQ-C30 Reference Values manual [34].

The nutrition facts questionnaire consists of 20 multiple-choice questions and each answer is marked in a range from 0 to 3. The total score is divided into four score profiles (‘poor’ 0–29, ‘fair’ 30–39, ‘good’ 40–49, ‘excellent’ 50–60) and, in addition to this classification, three scales are considered: ‘lifestyle and healthy eating knowledge’, ‘nutritional and cancer knowledge’ and ‘food habits’. These scales were constructed summing up the scores from questions belonging to every scale topic. Scores were then rescaled with a proportion from 0 to 100, so that the higher the score is (near to 100), the better the knowledge is. There are no existing reference data, since the questionnaire was developed by the study team expressly for this project.

## Results and discussion

Supporto Nutrizionale study was designed to help patients with cancer to deal with information on nutritional care, to offer a space where to interact with experts and/or others participants, and this would positively affect the patients’ knowledge, the quality of life and the psychological status when compared to controls.

**Figure 4:**
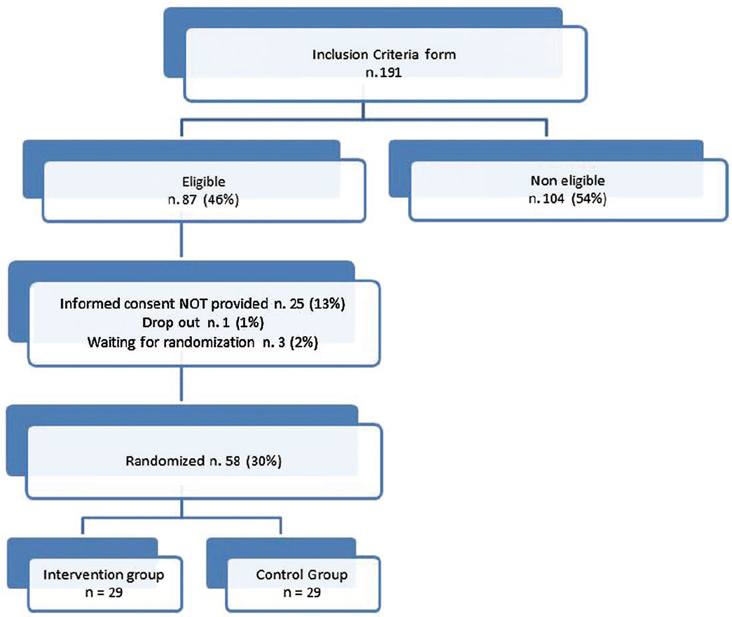
Supporto Nutrizionale study: participation flow after 6 months.

The study recruitment started on 1 March and it is still open. After 6 months of recruitment, 191 subjects compiled the Inclusion Criteria form and only 58 (30%) have been randomized as shown in the participation flow (Figure 4). The signed informed consent form has not been sent by 25 subjects (13%) and 104 subjects (54%) resulted not eligible for the study. The main reasons for exclusion are reported in Table 2. A significant weight loss and receiving palliative care are the most recorded reasons for exclusion accounting for 51%. The high exclusion rate is probably due to the restrictive exclusion criteria that leave out advanced patients with cancer.

The baseline questionnaires have been filled by 50 out of 58 randomized subjects of the IG and CG. Socio-demographic characteristics of participants are presented in Table 3. Participants in both groups are mainly females (77% for IG, 88% for CG), married (69% for IG, 79% for CG) and have at least a high school education level (42% for IG, 58% for CG) as reported by others study [9,35–37]. The mean age is 52, 2 years (SD± 9.9, range 32–70) for IG and 49, 7 (SD± 8.9, range 31–66) for CG. The medium body mass index [BMI; calculated as weight (kg)/ height (m)^2^] is 24, 1 for IG and 24, 0 for CG and respectively 19% and 26% experienced a weight loss. The study population, even if small, is in line with the results of other studies. Participants motivated to approach this intervention study are, as expected, well educated and their average age is 51,0 years (IG and CG) similarly to data reported by Eurostat [38] analysing the computer skills and internet use across all European countries.

The most common tumour site is breast (46% for IG and 75% for CG), followed by gastrointestinal tract (31% for IG, 13% for CG) and the time of diagnosis was in recent years, mainly from 2010 to 2011 for both groups (65% for IG, 63% for CG). Regarding clinical information, participants of the IG and CG reported to be treated mainly with chemotherapy (IG 35% and CG 33%) and other treatments (monoclonal antibodies, hormonal therapy, chemoembolization, etc) (IG 35% and CG 33%) and had surgery (IG 81% and CG 71%). The most common side effects of treatments are dry mouth and mouth soreness (50% for IG and 21% for CG), changes in taste (42% and 25%) and feeling full quickly (15% for IG and 33% for CG). Hypertension is the most common concomitant pathology (31% for IG and 8% for CG).

A cancer diagnosis is a major stressor and as a result, many patients experience a range of psychosocial difficulties, including depression, anxiety, loneliness, uncertainty and loss of control, and fears about cancer recurrence [39–41]. They search for support that could contribute to general well-being and that buffers the impact of stressful experiences [42]. In our study population, the prevalence of psychological distress (Table 4) is high, 27% in the IG and 33% in the CG, considering the 4 classes of scores at the baseline. PDI scores are high probably due to the short time from the diagnosis and their clinical status, since subjects are still under treatments. Considering the medium score the results are similar for IG and CG, respectively 30.6 and 31.2. In published studies, patients exhibit a high prevalence of psychological distress ranging from 13% to 29% at 1 or more years from the end of treatments [43]. Regarding QoL, results are shown in Table 5. These data collected at baseline show a low “role functioning” score for IG and “emotional functioning” and “social functioning” scores for both groups, while “fatigue” and “nausea and vomiting” respectively for IG and CG are the worsened symptoms compared with reference values. The result seems to be worsened than the reference data for both groups, but we expect a positive effect of this intervention study, nevertheless the stage of the disease is the major issue influencing the results. Results from the questionnaire on nutrition facts are shown in Table 6. As a result, the majority of participants showed a good-excellent score profile (65% for IG, 54% for CG) and the median score is 42, 1 for IG and 41, 0 for CG. Regarding the scales, “lifestyle and healthy eating knowledge” is the scale with the best results, while the worst results could be found in “nutritional and cancer knowledge” which consist of some questions about nutritional problems and their solution, where we expect the major improvement after the interventions jointly with an increase in the medium score.

**Figure 5:**
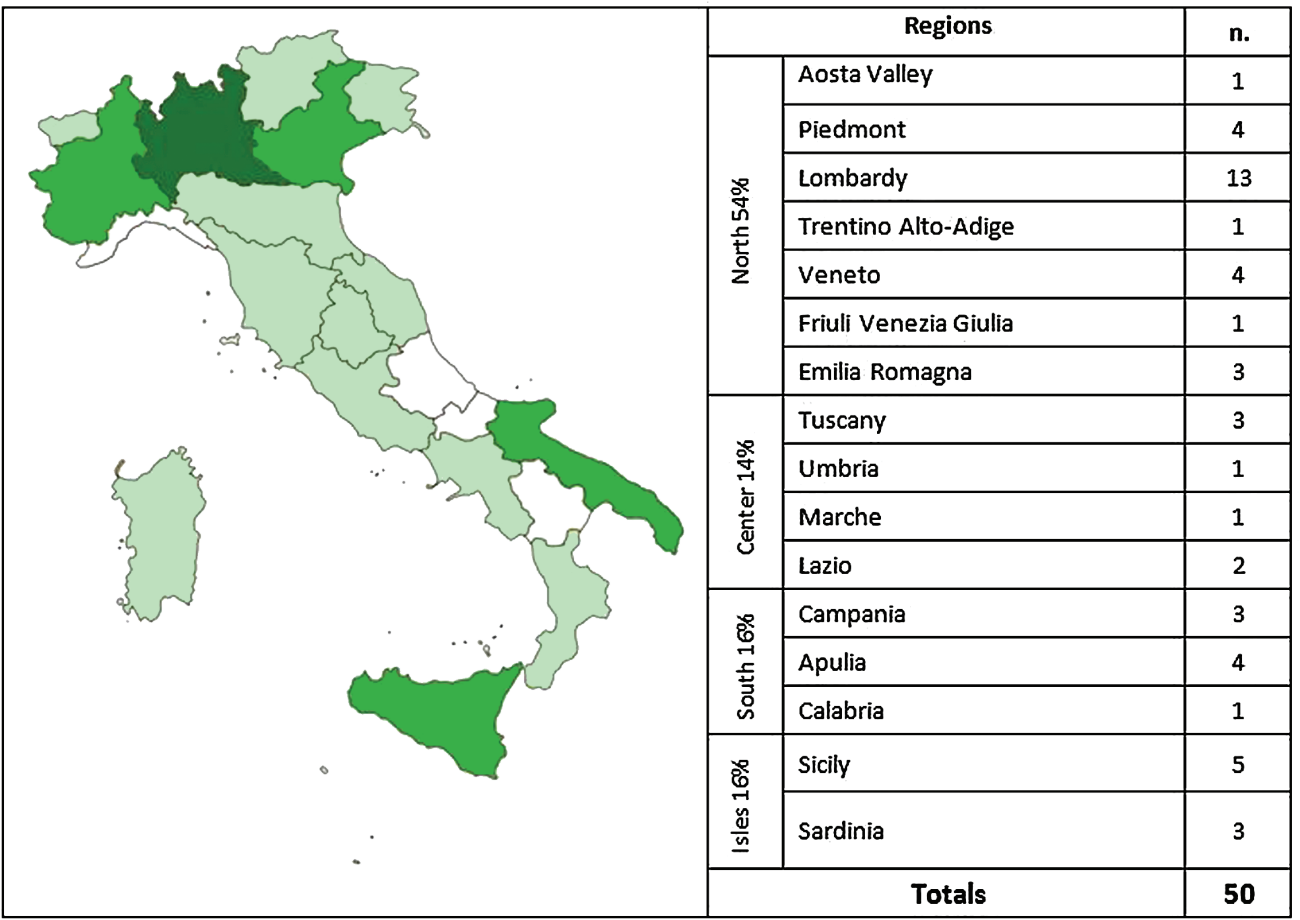
Subjects distribution on the Italian territory for intervention and control group (n. 50 participants).

Figure 5 shows the participants distribution according to residence on the Italian territory. The majority comes from North Italy (54% for IG and CG) and Lombardy is the most representative region (26% of total subjects).

From the beginning of the study, it has been registered a total of 48 actions during the first 4 months of intervention (Table 7), including votes to contents, comments and forum messages. Most viewed baseline (B) contents within the first 4 months of intervention have been “what is cancer and its therapies” and “cancer prevention recommendations”, while most viewed weekly (W) contents have been “nausea” and “constipation” (Table 8).

Strengths of this study include the use of a randomized design and the recruitment of participants from a nationwide public, with a good coverage of the Italian territory with subjects from 16 out of 20 Italian regions, open to all patients with cancer. Heterogeneity of cancer diagnosis, treatments and treatments stage are expected for this study and even if they may enlarge overall variability, they can improve the generalization of the findings and provide a more realistic reflection of the population of cancer survivors commonly using Internet.

Some of the methodological limitations of this study include a small sample size (at least concerning this preliminary results), lack of long-term follow-up measurements that can compromise confidence in the findings, and lack of any direct contact with the participants (self-report data).

## Conclusion

In Italy, cancer information is quite lacking in many settings as reported by De Lorenzo and colleagues [44]. Actually there are still patients who are not adequately informed regarding diagnosis, treatments and how to deal with symptoms. As reported in a recent AIMaC survey [45], patients with cancer after hospital discharge are looking for information on how to deal with nutrition during the disease for the 29%, which follows only to the first request regarding social security during the therapies and follow-up (61%).

The Internet is potentially a more powerful channel for delivering messages and information, improving access to expert care and feedback. This technology not only provides an opportunity to present information in a larger number of formats, but it also provides the greatest ability to enhance user’s interaction and understanding of the material. The Internet and the interactive programs in the care of patients with breast cancer were effective in increasing patients knowledge and useful in helping patients to make decisions about their care [10,46,47], but they cannot identify a clear effect on patients outcomes. Outcomes varied widely between studies due mainly to not standardized and homogeneous methodological measure [9,48].

The data presented in this paper are collected at the baseline and after 6 months of intervention, representing preliminary data. These data describe our study population that represents what expected in term of age, sex and educational level in an intervention study delivered by Internet. We cannot draw any conclusions at the moment. Research is required to evaluate the impact of the Internet to help, to inform and to support patients with cancer. To the best of our knowledge, no previous study has examined the effect of such intervention on how to manage the nutrition and eating problems rising during cancer symptoms. The results of this randomized intervention may help in the evaluation of the efficacy of these interventions in cancer setting.

## Figures and Tables

**Table 1:**
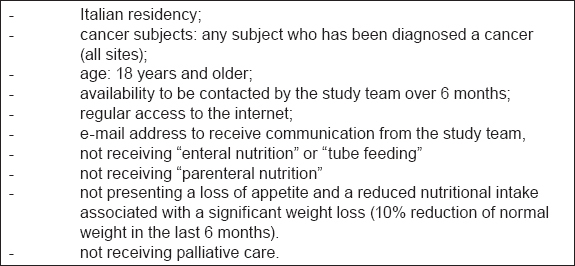
Inclusion criteria of the Supporto Nutrizionale study (2011)

**Table 2:**
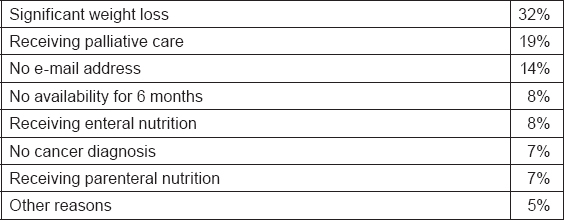
Reasons for exclusion from the Supporto Nutrizionale study after 6 months recruitment (March–September)

**Table 3:**
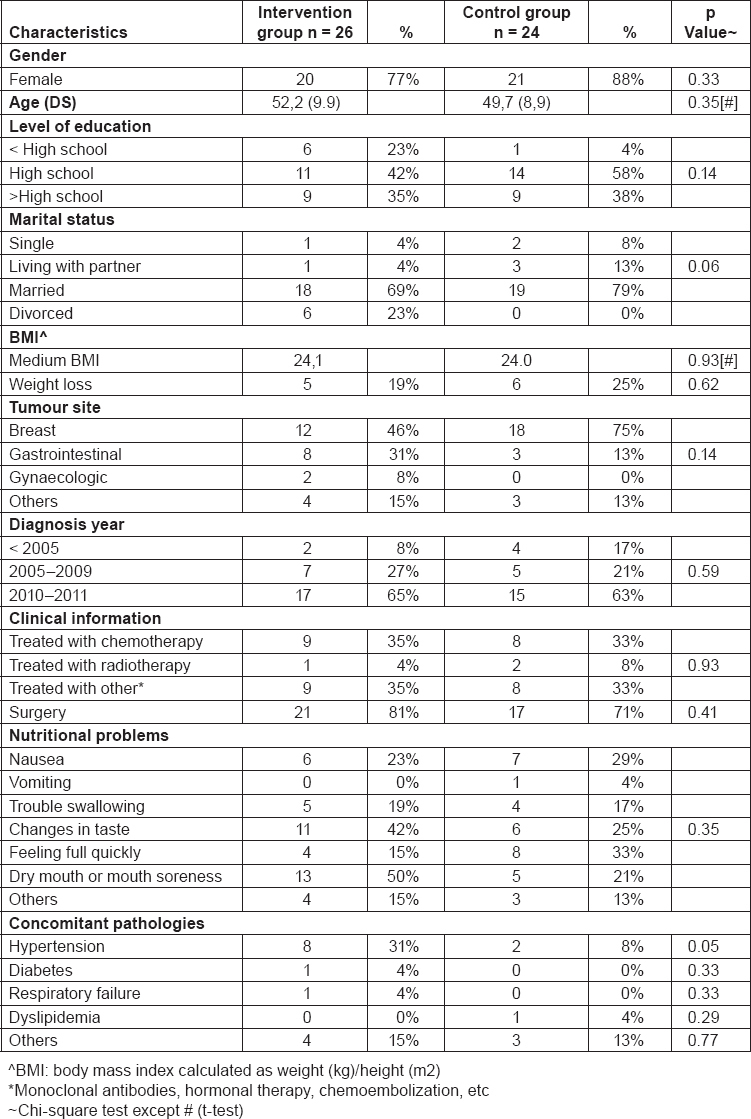
Socio-demographic characteristics at baseline for intervention group (IG) and control group (CG) (n. 50 randomized participants)

**Table 4:**
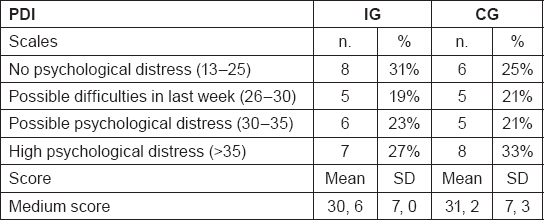
PDI results for the four different scales and the medium score at baseline for intervention group (IG) and control group (CG) (n. 50 randomized participants)

**Table 5:**
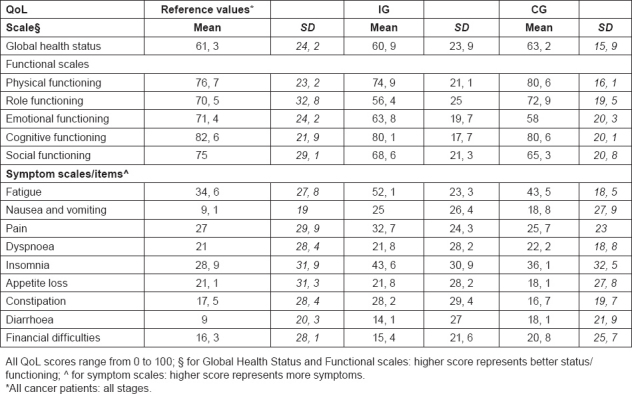
QoL results for intervention group (IG) and control group (CG) compared with references values at baseline (n. 50)

**Table 6:**
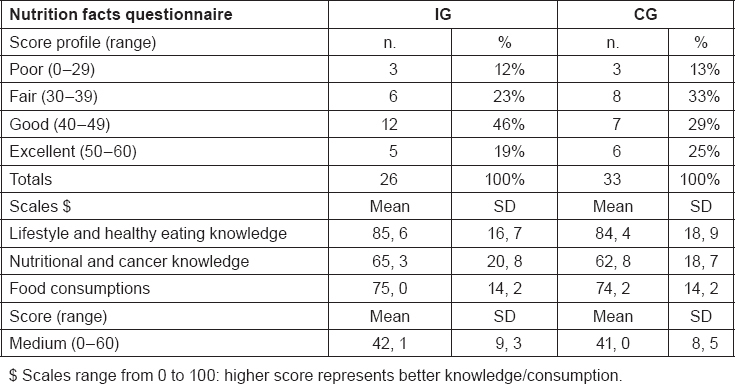
Nutrition facts questionnaire distribution of score profiles, scales and medium score for intervention group (IG) and control group (CG) at baseline (total n. 50 subjects)

**Table 7:**
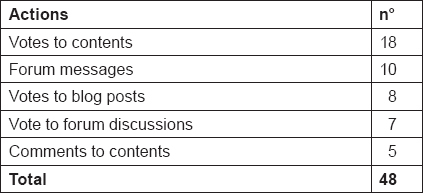
Total of actions in the website after 4 months of intervention for intervention group (n. 26)

**Table 8:**
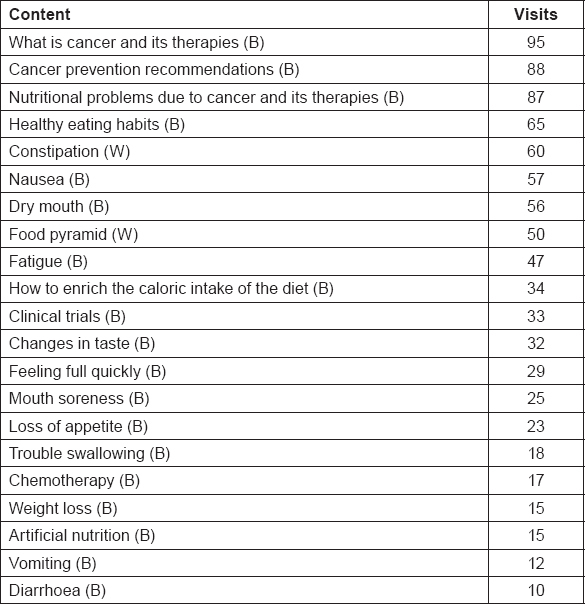
Most viewed baseline (B) and weekly (W) contents after 4 months of intervention for intervention group (n. 26)

## Appendix 1: Questionnaire on nutrition facts

1. Which are the principles of healthy eating?
  - Do not eat much and increase vitamins consumption
  - Follow a balanced diet using also minerals and vitamins supplements
  - Beware of weight, decrease fat, sugars and salt consumption, increase fruit and vegetables consumption
  - Privilege the proteins and fat consumption and complete with fruit and vegetable
  - Don’t know
2. What does a healthy lifestyle consist of?
  - Eat more fruit, vegetables, drink more water, decrease fat consumption, be more physically active
  - Be physically active, limit alcohol consumption, do not smoke and follow a healthy eating plan
  - Do not smoke, do not drink alcoholic beverages, be physically active
  - Do vigorous physical activity
  - Don’t know
3. What is the food pyramid?
  - A graphic where weekly suggested physical activity levels are placed in a decreasing order
  - A graphic that shows how many portions of every food groups have to be consumed per day
  - A guide for the population that suggests some recommendations on food to be consumed monthly
  - A tool to be used from people that want to follow a healthy vegetarian diet
  - Don’t know
4. Does a diet to fight cancer exist?
  - Yes
  - Yes, we need to add vitamins supplements
  - No, it doesn’t
  - No, it is necessary to follow a healthy diet
  - Don’t know
5. It is common to lose weight throughout the course of cancer?
  - Yes, always
  - Yes, it happens often
  - No, never
  - No, otherwise there could be a weight gain
  - Don’t know
6. Do I have to reduce physical activity throughout the course of cancer?
  - Yes, you’d better rest and lay in bed
  - Yes, you’d better rest
  - No, you’d better do even vigorous physical activity
  - No, you’d better do physical activity appropriated with your conditions (i.e. walk)
  - Don’t know
7. Can I use supplements?
  - Yes, always
  - Yes, but your physician should evaluate your conditions first
  - No, never
  - No, because supplements can cause some adverse effects
  - Don’t know
8. When Artificial Nutrition must be used?
  Artificial Nutrition is a practice that allows the feeding of those patients that, for many reasons, are not able to introduce, even partially, by mouth liquid or solid foods.
  - Always throughout cancer
  - When the patient is no more able to feed himself autonomously
  - It is not suggested
  - Never, it is suggested only in cases when oral feeding is dangerous
  - Don’t know
9. Are there food that can reduce collateral effects due to chemotherapy and radiotherapy?
  - Yes, fruit and vegetables
  - Yes, meat and fish
  - No, none in particularly
  - No, but a well nourished patient can better cope with the collateral effects
  - Don’t know
10. Can I avoid changes in taste during cancer treatments (e. g. chemotherapy), without mouth soreness?
  - Yes, with some tricks like using soy sauce, lemon juice, vinegar or chewing gums
  - Yes, eating bitter foods
  - No, because it seldom happens
  - No, because it is not possible to avoid changes in taste
  - Don’t know
11. If there is loss of appetite, what should I do?
  - Often drink water and carbonated drinks, eat many snacks
  - Eat many snacks, eat dry foods and always lay down to bed
  - Eat small and frequent meals, make food appetizing, eat slowly and rest after every meal
  - Cook meals by yourself, eat frequently, eat many snacks
  - Don’t know
12. What can I do to stop nausea and vomiting?
  - Eat according to one’s needs
  - Eat small and frequent meals, dry foods, do not introduce too many liquids
  - Do not eat anything and drink enough to stay hydrated
  - Eat fat foods and foods that slow down digestion
  - Don’t know
13. What can I do if I experience constipation?
  - Eat small and frequent foods, preferring high-protein foods (meat, fish and cheese) and drink frequently
  - Use vitamin and mineral supplements, drink frequently and practice vigorous physical activity
  - Add more fibre to the diet (fruit, vegetables, whole grain cereals) and help yourself with fruit syrups or juices, practice moderate physical activity
  - Address to a physician
  - Don’t know
14. What can I do if I have a trouble in swallowing foods?
  - Address to a physician
  - Drink frequently, suck ice cubes, prepare a soft diet using sauces and liquids (broth, gravy)
  - Eat small and frequent foods, drink frequently carbonated drinks, eat sweet and tasty foods
  - Eat high-calories foods and nutritional supplements, practice a moderate physical activity
  - Don’t know
15. What can I do if I have dry mouth?
  - Drink frequently, eat sweets and chocolate
  - Drink low alcoholic beverages to avoid dry mouth
  - Drink frequently and hydrate lips, do not have snacks
  - Drink frequently, suck ice cubes, eat soft foods, hydrate lips
  - Don’t know
16. What can I do if I have mouth soreness?
  - Eat small and frequent foods
  - Drink plenty of liquids, avoid salty, spicy and acid foods, eat soft foods
  - Eat many snacks, drink frequently, hydrate lips
  - Consume nutritional supplements, drink frequently, hydrate lips
  - Don’t know
  Next questions refer to your current eating habits. Please report consumptions that are closer to your current eating habits.
17. How many fruit and vegetable servings do you eat daily?
  - Less than 1 a day
  - 2–3 a day
  - 4–5 a day
  - More than 5 a day
18. How much meat do you eat weekly?
  [1 meat serving is 70–100 g (raw weight); please include in your count also cold cuts, 1 serving = 50 g]
  - Less than 2 servings per week
  - From 2 to 4 servings per week
  - From 4 to 8 servings per week
  - More than 8 servings per week
19. How many alcoholic units do you consume daily?
  [One alcoholic unit is 1 glass of wine, or 1 can of beer or in 1 shot of hard liquor (40 ml)]
  - I do not drink alcoholic drinks or less than 1 a day
  - 1–2 a day
  - 3–4 a day
  - More than 4 a day
20. Do you practice physical activity?
  - No, I don’t
  - Yes, I practice soft physical activity (walking, go up the stairs)
  - Yes, I practice medium intensity physical activity (go cycling, gardening, housework)
  - Yes, I practice vigorous physical activity (more than 3 hours a week of sports)

## References

[1] Eng TR, Maxfield A, Patrick K, Deering MJ, Ratzan SC, Gustafson DH (1998). Access to health information and support: a public highway or a private road?. JAMA.

[2] Powell J, Clarke A (2002). The WWW of the World Wide Web: Who, What, and Why?. J Med Internet Res.

[3] Santoro E (2009). Web 2.0 e Medicina.

[4] Chou WY, Hunt YM, Beckjord EB, Moser RP, Hesse BW (2009). Social media use in the United States: implications for health communication. J Med Internet Res.

[5] Patrick K (1999). How patients use the web for second opinions. West J Med.

[6] Williams P, Nicholas D, Huntington P, McLean F (2002). Surfing for health: user evaluation of a health information website. Part one: Background and literature review. Health Info Libr J.

[7] Eysenbach G (2003). The impact of the Internet on cancer outcomes. CA Cancer J Clin.

[8] Peterson G, Aslani P, Williams KA (2003). How do consumers search for and appraise information on medicines on the Internet? A qualitative study using focus groups. J Med Internet Res.

[9] Hoey LM, Ieropoli SC, White VM, Jefford M (2008). Systematic review of peer-support programs for people with cancer. Patient Educ Couns.

[10] Gustafson DH, Hawkins R, Pingree S, McTavish F, Arora NK, Mendenhall J, Cella DF, Serlin RC, Apantaku FM, Stewart J, Salner A (2001). Effect of computer support on younger women with breast cancer. J Gen Intern Med.

[11] Winzelberg AJ, Classen C, Alpers GW, Roberts H, Koopman C, Adams RE, Ernst H, Dev P, Taylor CB (2003). Evaluation of an internet support group for women with primary breast cancer. Cancer.

[12] Owen JE, Klapow JC, Roth DL, Shuster JL, Bellis J, Meredith R, Tucker DC (2005). Randomized pilot of a self-guided internet coping group for women with early-stage breast cancer. Ann Behav Med.

[13] Kroeze W, Werkman A, Brug J (2006). A systematic review of randomized trials on the effectiveness of computer-tailored education on physical activity and dietary behaviors. Ann Behav Med.

[14] Norman GJ, Zabinski MF, Adams MA, Rosenberg DE, Yaroch AL, Atienza AA (2008). A review for eHealth interventions for physical activity and dietary behaviour change. A J Prev Med.

[15] Joint WHO/FAO Expert Consultation (2003). Diet, nutrition and the prevention of chronic diseases.

[16] Aaronson NK, Cull A, Kaasa S, Sprangers M (1994). The EORTC modular approach to Quality of Life assessment in Oncology. Int J Ment Health.

[17] Sobotka L (2004). Nutritional support in severe malnutrition in basics in clinical nutrition.

[18] Thomas R, Davies N (2007). Lifestyle during and after cancer treatment. Clin Oncol (R Coll Radiol).

[19] Wojtaszek CA, Kochis LM, Cunningham RS (2002). Nutrition impact symptoms in the oncology patient. Oncology.

[20] **EuroCancerComs** funded under the EU FP7 “Science in Society”. www.eurocancercoms.eu.

[21] Muscaritoli M, Preziosa I, Canelli A, De Leo S, Associazione Italiana Malati di Cancro (2008). La nutrizione del malato oncologico.

[22] Rossi Fanelli F, Muscaritoli M, Laviano A, Gavazzi C, Federazione Italiana delle Associazioni di Volontariato in Oncologia (2006). Neoplasia e perdita di peso Che cosa fare?.

[23] De Lorenzo F, Gnagnarella P, McVie JG, Del Campo L, Oricchio R (2011). Contributo Italiano al Progetto Europeo Eurocancercoms (Supporto nutrizionale in soggetti con cancro: un sito internet dedicato).

[24] Misotti AM, McVie JG, Milolidakis G, De Lorenzo F, Santoro L, Gnagnarella P (2011). Supporto Nutrizionale in soggetti con cancro: un sito Internet dedicato.

[25] American Cancer Society (2010). Nutrition for the Person With Cancer During Treatment: A Guide for Patients and Families.

[26] National Cancer Institute (2009). Eating hints: Before, During and After Cancer Treatment.

[27] Ministero delle Politiche Agricole e Forestali, Istituto Nazionale di Ricerca per gli Alimenti e la Nutrizione (2003). Linee guida per una sana alimentazione italiana.

[28] World Cancer Research Fund and American Institute for Cancer Research (2007). Food, Nutrition, Physical Activity, and the Prevention of Cancer: a Global Perspective.

[29] Istituto Europeo di Oncologia (2002). La Nutrizione durante il trattamento Oncologico.

[30] Morasso G, Costantini M, Baracco G, Borreani C, Capelli M (1996). Assessing psychological distress in cancer patients: validation of a self-administered questionnaire. Oncology.

[31] Aaronson NK, Ahmedzai S, Bergman B, Bullinger M, Cull A, Duez NJ, Filiberti A, Flechtner H, Fleishman SB, de Haes JC (1993). The European Organization for Research and Treatment of Cancer QLQ-C30: a quality-of-life instrument for use in international clinical trials in oncology. J Natl Cancer Inst.

[32] Morasso G (2004). PsicologicalDistress Inventory (PDI) Manuale. Servizio di Psicologia. Istituto Nazionale per la ricerca sul cancro di Genova.

[33] Fayers PM, Aaronson NK, Bjordal K, Groenvold M, Curran D, Bottomley A on behalf of the EORTC Quality of Life Group (2001). The EORTC QLQ-C30 Scoring Manual.

[34] Scott NW, Fayers PM, Aaronson NK, Bottomley A, de Graeff A, Groenvold M, Gundy C, Koller M, Petersen MA, Sprangers MAG, on behalf of the EORTC Quality of Life Group (2008). EORTC QLQ-C30 Reference Values.

[35] Eysenbach G, Powell J, Kuss O, Sa ER (2002). Empirical studies assessing the quality of health information for consumers on the world wide web: a systematic review. JAMA.

[36] Mayer DK, Terrin NC, Kreps GL, Menon U, McCance K, Parsons SK, Mooney KH (2007). Cancer survivors information seeking behaviors: a comparison of survivors who do and do not seek information about cancer. Patient Educ Couns.

[37] Høybye MT, Dalton SO, Deltour I, Bidstrup PE, Frederiksen K, Johansen C (2010). Effect of Internet peer-support groups on psychosocial adjustment to cancer: a randomised study. Br J Cancer.

[38] Demunter C, European Commission, Eurostat (2006). How skilled are Europeans in using computers and the internet? Statistics in focus17. http://epp.eurostat.ec.europa.eu/cache/ITY_OFFPUB/KS-NP-06-017/EN/KS-NP-06-017-EN.PDF.

[39] Cliff AM, MacDonagh RP (2000). Psychosocial morbidity in prostate cancer: II. A comparison of patients and partners. BJU Int.

[40] Mullen P, Smith R, Hill E (1993). Sense of coherence as a mediator of stress for cancer patients and spouses. J Psychosoc Oncol.

[41] Dunn J, Steginga S, Rosoman N, Millichap D (2003). Review of peer support in the context of cancer. J Psychosoc Oncol.

[42] Cutrona CE (1990). Stress and social support: in search of optimal matching. J Soc Clin Psychol.

[43] Morasso G, Di Leo S, Caruso A, Decensi A, Beccaro M, Berretta L, Bongiorno L, Cosimelli M, Finelli S, Rondanina G, Santoni W, Stigliano V, Costantini M (2010). Evaluation of a screening programme for psychological distress in cancer survivors. Support Care Cancer.

[44] De Lorenzo F, Ballatori E, Di Costanzo F, Giacalone A, Ruggeri B, Tirelli U (2004). Improving information to Italian cancer patients: results of a randomized study. Ann Oncol.

[45] Alleanza Contro il Cancro, Associazione Italiana Malati di Cancro, Istituto Superiore di Sanità (2009). Indagine su cancro e informazione: che cosa chiedono i malati.

[46] Davison BJ, Degner LF (2002). Feasibility of using a computer-assisted intervention to enhance the way women with breast cancer communicate with their physicians. Cancer Nurs.

[47] Austoker J, Bankhead C, Forbes LJ, Atkins L, Martin F, Robb K, Wardle J, Ramirez AJ (2009). Interventions to promote cancer awareness and early presentation: systematic review. Br J Cancer.

[48] Ryhänen AM, Siekkinen M, Rankinen S, Korvenranta H, Leino-Kilpi H (2010). The effects of Internet or interactive computer-based patient education in the field of breast cancer: a systematic literature review. Patient Educ Couns.

